# Telemonitoring type 1 diabetes patients during the COVID-19 pandemic in Brazil: was it useful?

**DOI:** 10.20945/2359-3997000000309

**Published:** 2020-11-09

**Authors:** Alessandra Saldanha de Mattos Matheus, Carolina Alves Cabizuca, Lucianne Righetti Monteiro Tannus, Aline Camin Passos, Amábile Cristyne Schmidt, Ana Tarasiuk de Gouveia, Bruno Moraes de Albuquerque Pessoa, Felipe Cerqueira Matheus, Gabriela Yea-Huey Yang, Josimara Araujo da Silva Divino, Juliana Affonso Mathiles, Juliana Leite Teixeira, Luiza de Souza Barroso, Max Benicio da Fonseca de Brito, Paula Melichar Suassuna, Roberta Arnoldi Cobas

**Affiliations:** 1 Universidade do Estado do Rio de Janeiro Policlínica Piquet Carneiro Unidade de Diabetes Rio de Janeiro RJ Brasil Unidade de Diabetes, Policlínica Piquet Carneiro, Universidade do Estado do Rio de Janeiro, Rio de Janeiro, RJ, Brasil; 2 Universidade do Estado do Rio de Janeiro Faculdade de Ciências Médicas Rio de Janeiro RJ Brasil Faculdade de Ciências Médicas, Universidade do Estado do Rio de Janeiro, Rio de Janeiro, RJ, Brasil

**Keywords:** Type 1 diabetes, COVID-19, telemonitoring

## Abstract

**Objectives:**

To evaluate the performance of telemonitoring in detecting clinical and psychological needs and adherence to the protective measures imposed by the COVID-19 pandemic in addition to providing remote assistance for patients with type 1 diabetes (T1D) in a public university center in Brazil.

**Subjects and methods:**

Telemonitoring protocol included phone calls and e-mails. Patients were asked to rate COVID-19-like symptoms, psychological symptoms, epidemiological issues, and adherence to diabetes management (insulin, exercise, and diet) using a 0-to-10 scale. An e-mail address and phone number were offered for further contact if needed. Clinical, demographic, and laboratorial data from the consultations before the pandemic were collected from medical records.

**Results:**

Among 321 patients with a previously scheduled consultation over the first 15 weeks of social distancing, 237 (73.8%) could be successfully contacted. Of these, 207 (87.3%) were exclusively evaluated by telemonitoring (190 only by phone or text message and 17 who were also reached by email), and 30 (12.7%) patients attended the consultation for medical reasons detected during the telephone screening. Overall, 44 (18.5%) patients reported COVID-19-like symptoms. One (2.3%) patient was hospitalized and subsequently died. Psychological symptoms were reported by 137 (60.4%) patients and 30 (12.7%) required remote psychological assistance. Appropriate social distancing was performed by 203 (87.9%) patients, and 221 (97.8%) referred use of masks.

**Conclusions:**

Telemonitoring T1D patients during the pandemic helped reduce the need for in-person consultations, detect clinical and psychological needs, and offer support to patients in addition to monitoring suspected COVID-19 cases and the adherence to protective measures.

## INTRODUCTION

**D**iabetes and poor glycemic control are risk factors for adverse outcomes of COVID-19, such as severe acute respiratory syndrome (SARS) and death (1,2). In a multicenter study including type 1 diabetes (T1D) patients with confirmed or suspected COVID-19, 17.5% required hospital admission, and 22.2% required intensive care, and one-third experienced diabetic ketoacidosis. These results reinforce the importance of closely monitoring patients with T1D during the COVID-19 pandemic (3).

The International Diabetes Federation (4) and the Brazilian Diabetes Society (5) published specific recommendations for this period, such as self-monitoring blood glucose to avoid hyper- or hypoglycemia, guidance about COVID-19 symptoms, awareness of immediate medical evaluation, and assurance of a suitable stock of medications and supplies. However, the multidisciplinary therapeutic approach usually recommended for diabetes care was no longer feasible during the COVID-19 pandemic due to the determination of social distancing. Therefore, the replacement of the usual in-person consultations with remote monitoring became urgently needed, leading to changes in many regulatory rules to telehealth (6) and unprecedented interactions among health care providers and patients (7).

In accordance with the demands imposed by the pandemic, the medical team at the Rio de Janeiro State University's Diabetes Unit instituted a telemonitoring protocol to manage patients with T1D, thereby minimizing their exposure risk.

The aim of this study was to evaluate the performance of telemonitoring in detecting clinical and psychological needs and adherence to the protective measures imposed by the COVID-19 pandemic in addition to providing remote assistance for patients with T1D in a public university center in Brazil.

## SUBJECTS AND METHODS

This observational study was conducted at the Diabetes Unit of Rio de Janeiro State University during the COVID-19 pandemic. The study was approved by the center's local ethics committee, and *Plataforma Brasil's Certificado de Apresentação para Apreciação Ética* (CAEE) number was 31780320.3.0000.5259.

Outpatients with T1D (defined by American Diabetes Association criteria) receiving follow-up treatment at our unit who had an appointment scheduled for the pandemic period (March to June 2020) were eligible for telemonitoring. We excluded those whom we did not successfully contact from further analysis because telemonitoring was not feasible.

Clinical, demographic, and laboratorial data recorded in the last consultation before the pandemic were collected from medical records: age, duration of diabetes, body mass index (BMI), diagnosis of hypertension, diabetic retinopathy, diabetic neuropathy, and glomerular filtration rate (estimated by the Chronic Kidney Disease Epidemiology Collaboration creatinine equation in adults (8) or the Schwartz equation (9) for children and adolescents < 18 years). Patients were classified as children (younger than 19 years old), adults (between 19 and 59 years old), and older adults (60 years old or older). Renal dysfunction was considered a glomerular filtration rate less than 60 ml/min/ 1.73 m^2^. Patients were considered overweight or obese if BMI ≥ 25 or ≥ 30 kg/m^2^ in adults, and ≥ 85th and ≥ 95th percentile in subjects < 18 years old, respectively.

The information assessed through telephone calls was as follows:

1. Clinical data: presence of symptoms suggestive of COVID-19 (fever, cough, headache, anosmia, loss of taste, myalgia, and shortness of breath, as well as fatigue and gastrointestinal symptoms); availability of tests for diagnostic confirmation; and hospitalization or death.
2. Epidemiological data: close contact with individuals suspected or confirmed to have COVID-19, adherence to social distancing and mask recommendations, and influenza vaccination. Social distancing was classified as total if the patients remained at home all the time, partial if the patients left the house only for essential activities of short duration, and absent if they left home for work or nonessential activities. Appropriate use of masks was considered when the recommendation to use them outside home was respected.
3. Difficulties in acquisition of supplies for diabetes treatment.
4. Adherence to diabetes management: taking medication (0 to 10 scale), diet (0 to 10 scale), and self-reported frequency of physical exercise.
5. Psychological effects of the COVID-19 pandemic: presence of symptoms such as anxiety, fear, and insomnia, as well as the need for support by a mental health professional.

### Statistical analysis

The statistical analysis was performed using SPSS version 25.0.

Nonparametric data are presented as median values (interquartile range). Mann-Whitney test was used for comparison between variables with nonparametric distribution and chi-square test was used to compare frequencies. A two-sided *P* value < 0.05 was considered statistically significant.

## RESULTS

**Figure 1:**
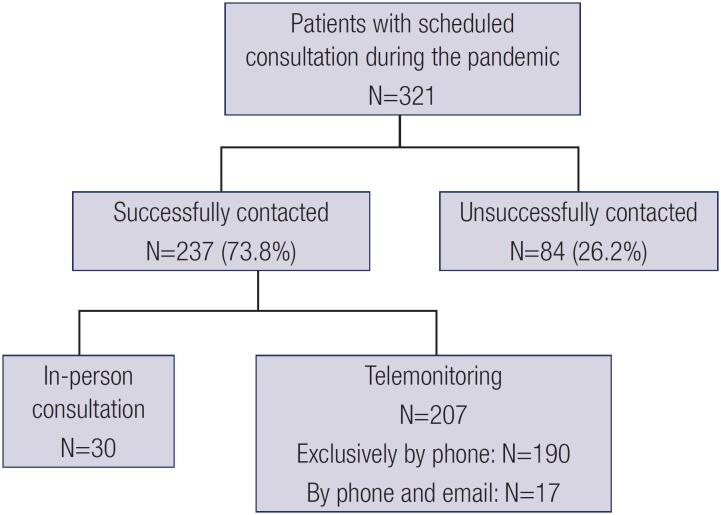
Flowchart showing the performance of the telemonitoring strategy

From 321 T1D patients with previously scheduled appointments for the period of COVID-19 pandemic, 237 (73.8%) could be successfully contacted by phone. For 84 (26.2%) patients, telemonitoring was not possible because of wrong phone numbers in the register or the patients did not answer the call despite multiple attempts. Of the 237 patients contacted by phone, 207 (87.3%) were exclusively evaluated by telemonitoring (190 only by phone or text message and 17 who were also reached also by email), and 30 (12.7%) patients attended consultations for medical reasons detected during the telephone screening (Figure 1).

No differences were observed in clinical and demographic data between patients successfully and unsuccessfully contacted for telemonitoring (data not shown).

Among successfully contacted patients, the majority were adults (63.7%) with a diabetes duration of 12 [5.5-22] years and A1c of 8.7% [7.7-10]. Clinical and anthropometric characteristics are presented in Table 1.

**Table 1:** Clinical and epidemiological data from the whole population and comparison between symptomatic and asymptomatic patients

		Total	COVID-19-likesymptomatics	Asymptomatics	P value
N		237	44	193	
Gender (Female)		129 (54.4)	28 (63.6)	101 (52.3)	0.174
Age (years)		27 [16-43]	33 [18.5-43]	26 [16-43]	0.532
Pediatric		74 (31.2)	11 (25)	63 (32.6)	0.334
Adults		151 (63.7)	32 (72.7)	119 (61.7)	
Elderly		12 (5.1)	1 (2.3)	11 (5.7)	
Duration of diabetes (years)		12 [5.5-22]	16 [7.5-23.5]	12 [5-22]	0.255
HbA1c (%)		8.7 [7.7-10]	8.6 [7.7-9.5]	8.7 [7.6-10.2]	0.637
Renal dysfunction^1^		16 (9.2)	1 (3.2)	15 (10.6)	0.201
Retinopathy^2^		70 (40.5)	12 (40)	58 (40.6)	0.955
Neuropathy^3^		30 (18.3)	8 (25)	22 (16.7)	0.274
Body mass index^4^		23.4 [19.7-27.1]	24.5 [21.2-28.6]	23.3 [19.7-26.9]	0.428
Eutrophic		135 (57.2)	23 (52.3)	112 (58.3)	0.762
Overweight		68 (28.8)	14 (31.8)	54 (28.1)	
Obesity		33 (14)	7 (15.9)	26 (13.5)	
Hypertension^5^		53 (22.8)	10 (22.7)	43 (22.9)	0.984
Frequency of symptoms					
Fever			18 (40.9)		
Myalgia			16 (36.4)		
Headache			15 (34.1)		
Cough			14 (31.8)		
Dysgeusia			12 (27.3)		
Anosmia			9 (20.5)		
Shortness of breath			9 (20.5)		
Others			12 (27.3)		
Hospitalization			1 (2.3)		
Death			1 (2.3)		
Practice of exercise^6^		62 (27.4)	10 (23.3)	52 (28.4)	0.495
Adherence to diet^7^		7 [5.8-8]	7 [6-8]	7 [5-8]	0.616
Adherence to treatment^8^		137 (60.4)	9 [8-10]	10 [8-10]	0.174
Psychological symptoms^9^		137 (60.4)	28 (65.1)	109 (59.2)	0.478
Psychological consultation required		30 (12.7)	11 (25)	19 (9.8)	0.006
In-person consultation		30 (12.7)	4 (9.1)	26 (13.5)	0.430
Telemonitoring		207 (87.3)	40 (90.9)	167 (86.5)	0.43066
Influenza Vaccine^10^		128 (56.1)	23 (53.5)	105 (56.8)	0.697
Contact with confirmed or suspected COVID-19^11^		37 (15.8)	16 (38.1)	21 (10.9)	0.000
Social distancing^12^					
	None	28 (12.1)	6 (14)	22 (11.7)	0.431
	Total	136 (58.9)	28 (65.1)	108 (57.4)	
	Partial	67 (29)	9 (20.9)	58 (30.9)	
Use of masks^13^		221 (97.8)	42 (97.7)	179 (97.8)	0.955

Data presented as n (%) or median [interquartile range]. Missing data:

1 8 patients;

2 8 patients;

3 17 patients;

4 1 patient;

5 5 patients;

6 11 patients;

7 11 patients;

8 13 patients;

9 10 patients;

10 9 patients;

11 3 patients;

12 6 patients;

13 11 patients.

Data from chronic complications were obtained only for patients with criteria for annual screening.

Overall, 44 (18.5%) patients described COVID-19-like symptoms. Of those, 25% were pediatric patients, 72.7% were adults, and 2.3% elderly people. One patient was hospitalized and died (2.3%) during this period (Table 1). It is important to mention that she had T1D for 14 years, cardiac autonomic, peripheral neuropathy and retinopathy with a poor glycemic control (last A1c was 12.3%), and whose BMI was 18 kg/m^2^.

Considering the type of COVID-19-like symptom, fever (40.9%), myalgia (36.4%), headache (34.1%), and cough (31.8%) were the most prevalent. Other symptoms included loss of taste (27.3%), loss of smell (20.5%), shortness of breath (20.5%), and others (27.3%). Whether patients presented symptoms or not, no difference in sex, age, duration of diabetes, A1c levels, or presence of chronic complications was observed between them. Epidemiological data and adherence to preventive measures were also not different (Table 1).

Thirty (12.7%) patients required psychological attention performed through telemedicine, mostly in the symptomatic group (11 [25%] *vs.* 19)9.8%], *P* = 0.006; Table 1) The most prevalent psychological symptoms attributable to the COVID-19 pandemic were fear, insomnia, and anxiety, affecting 39.4%, 29.6%, and 46.3% of patients, respectively. The presence of psychological symptoms was not associated with gender, duration of diabetes, A1c levels, BMI, or adherence to insulin treatment or diet. Patients with psychological symptoms were older (30 [18-44] *vs.* 21.5 [15-38.5] years old, *P* = 0.044) and practiced physical exercise less frequently (29 [21.2%] *vs.* 33 [37.1%], *P* = 0.009). In addition, the adherence to diet was greater in patients who also adhered to physical activity (7.75 [6-9] *vs.* 7 [5-8], *P* = 0.012).

A higher proportion of pediatric and older patients respected social distancing (95.9% and 91.6%, respectively) compared to adults (83.5%, *P* = 0.001). Of those not completely social distancing, the majority in all age groups said they used masks outside. The adherence to influenza vaccination was higher in the older group (83.3%) compared to the adult (58.6%) and pediatric groups (46.5%, *P* = 0.036; Table 2)

**Table 2:** Clinical and epidemiological data from the whole population and stratification by age groups

		Pediatric population (n = 74)	Adults (n = 151)	Elderly(n = 12)	P value
Close contact with suspected or confirmed case^0^		8 (11.1)	28 (18.7)	1 (8.3)	0.270
Social distancing^1^					
	None	3 (4.1)	24 (16.4)	1 (8.3)	
	Total	61 (83.6)	71 (48.6)	4 (33.3)	
	Partial	9 (12.3)	51 (34.9)	7 (58.3)	0.001
Use of masks, yes^2^		69 (98.6)	140 (97.2)	12 (100)	0.711
Influenza vaccination, yes^3^		33 (46.5)	85 (58.6)	10 (83.3)	0.036
Adherence to diet^4^		7 [5-9]	7 [6-8]	6 [5-8]	0.699
Adherence to insulin treatment^5^		10 [9-10]	9 [8-10]	10 [8-10]	0.004
Physical exercise, yes^6^		17 (23.9)	43 (29.9)	2 (18.2)	0.513
Problems in supplies acquisition, yes^7^		9 (12.9)	27 (18.8)	3 (27.3)	0.379
Psychological symptoms^8^		35 (48.6)	95 (66)	7 (63.6)	0.047
Psychologic telemonitoring required		7 (9.5)	21 (13.9)	2 (16.9)	0.585

Missing data:

0 3 patients;

1 6 patients;

2 11 patients;

3 9 patients;

4 11 patients;

5 13 patients;

6 11 patients;

7 12 patients;

8 10 patients.

The effect of the pandemic on diabetes treatment stratified by age groups is presented in Table 2. Overall, 17.3% of the patients referred to difficulties acquiring medical supplies. The self-reported scores for adherence to diet and exercise frequency were similar among age groups. The self-reported score for insulin treatment adherence was different among age groups (10 [9-10] in pediatric cases vs 9 [8-10] in adult and 10 [8-10] in older person's cases, *P* = 0.004).

## DISCUSSION

During the COVID-19 pandemic, telemonitoring was expected to play an important role in assisting patients with chronic diseases to avoid their exposure to SARS-CoV-2 infection. However, the success of this strategy in monitoring T1D patients in the public health system in Brazil was unknown, as was the effect of the pandemic on patients’ physical and mental health. To fill this gap, our study showed that telemonitoring reached 74% of T1D patients followed-up at our diabetes unit during the COVID-19 pandemic, and it allowed us to select urgent cases requiring in-person consultation and provide treatment guidance and psychological support to reinforce protective measures. Brazil's health care system is traditionally structured on the model of face-to-face interactions between patients and clinicians, and this paradigm shift, especially under an urgent circumstance, was a major challenge.

To ratify our expectations, a 2015 Cochrane systematic review has already shown that telemedicine can result in improved blood glucose control in patients with diabetes (10). Similarly, children with asthma seen by telemedicine or in-person visits can achieve comparable degrees of asthma control (11). A recent Brazilian publication proposed that telehealth offers capabilities for remote screening, care, and treatment, and it assists monitoring, surveillance, detection, prevention, and mitigation of the effects on health care indirectly related to COVID-19 (12).

The prevalence of symptoms suggestive of COVID-19 in our population was 18.8%. Although the association with worse outcomes in patients with diabetes is well established (2), the susceptibility to SARS-CoV-2 infection does not appear to be increased in these patients (13,14). Thus, it is expected that infection rate among people with diabetes does not differ from the general population. Although none of the suspected COVID-19 cases could be confirmed due to lack of testing, almost all patients presented mild to moderate symptoms and one needed hospitalization and later died in this period. The absence of diagnostic confirmation can be explained by the low access to diagnostic tests because the public health strategy in Brazil was to test only severe cases of the disease. In fact, other respiratory viruses can be responsible for infections during this period (15) and data from the Brazilian Ministry of Health showed that COVID-19 was responsible for 53% of cases of hospitalization for SARS and 47% were due to other causes (16).

The T1D patients’ adherence to social distancing was high: 95.9%, 83.6%, and 91.7% of pediatric, adult, and older patients, respectively. In addition, almost all patients who did not perform distancing or did so partially adhered to the use of masks. According to data obtained by the technology company In Loco using a methodology for monitoring cellular signals, our findings were higher than the 40.1% social distancing rate observed in the State of Rio de Janeiro on June 23, 2020, and the 37.8% rate in Brazil on the same day. The maximum distancing rate recorded for the population in Rio de Janeiro since the beginning of lockdown was 64.1% on March 22, 2020 (17).

The COVID-19 pandemic may be associated with the onset or worsening of people's psychological disorders (18). The reduced contact with friends, familial support, and social network imposed by social distancing can trigger anxiety, fear, and sleep disorders. Particularly in patients with chronic diseases, including diabetes, these psychological symptoms may worsen due to the fear of clinical decompensation and adverse outcomes associated with COVID-19. The majority of our patients reported symptoms such as feeling anxious, feeling fearful, or difficulty sleeping, regardless of the presence of COVID-19-like symptoms. However, a greater number of COVID-19-like symptomatic patients asked for psychological support. According to a recent study based on telemedicine, 88.4% of older type 2 diabetes patients felt anxiety related to the current pandemic (19).

We observed a low adherence to physical exercise, which could be explained by the difficulty of exercising at home. However, studies prior to the COVID-19 pandemic have already shown that physical activity was performed by only 34% of patients with diabetes and the main reasons for inactivity included the perception of difficulty in exercising, feeling tired, and lack of time or local facilities (20,21). In addition, the score given to diet was lower than that attributed to the use of insulin in our study. Although dietary recommendations are important for optimal self-management in T1D patients, data show that this is one of the most difficult treatment pillars to follow (21).

Our study has some limitations. First, we were not able to contact all patients scheduled for consultations during the pandemic, which could have led to underestimation of the number of symptomatic patients and the number of hospitalizations or deaths. Furthermore, no symptomatic patient had a confirmatory test for COVID-19, so we cannot assure the etiology of flu-like syndrome.

To overcome the barriers to providing telemonitoring that we encountered, periodic updates of personal information in medical records must be performed. Moreover, considering that a multidisciplinary approach is essential for good metabolic control, supplementary to medical and psychological support, we propose that strategies be developed to provide nutritional guidance and physical exercise programs that can be easily performed at home.

In summary, telemonitoring T1D patients from our public diabetes center during the COVID-19 pandemic was useful to reduce the need for in-person consultations and to detect clinical and psychological needs while providing remote assistance for patients. In addition, phone contact allowed us to reinforce the recommended protective measures to minimize risk of SARS-CoV-2 exposure.
